# Editorial for the Special Issue “Sensing-Based Biomedical Communication and Intelligent Identification for Healthcare”

**DOI:** 10.3390/s24051403

**Published:** 2024-02-22

**Authors:** Wenyan Jia, Yi Gao, Zhi-Hong Mao, Mingui Sun

**Affiliations:** 1 Department of Electrical and Computer Engineering, University of Pittsburgh, Pittsburgh, PA 15260, USA; zhm4@pitt.edu (Z.-H.M.); drsun@pitt.edu (M.S.); 2 School of Biomedical Engineering, Shenzhen University Medical School, Shenzhen University, Shenzhen 518060, China; gaoyi@szu.edu.cn; 3 Department of Bioengineering, University of Pittsburgh, Pittsburgh, PA 15260, USA; 4 Department of Neurosurgery, University of Pittsburgh, Pittsburgh, PA 15260, USA

The integration of sensor technology in healthcare has become crucial for disease diagnosis and treatment. In the past decade, sensors of different types (e.g., electronic, optical, biological, and chemical sensors) and associated data processing algorithms have been developed in this field. Examples include noninvasive and minimally invasive sensors that monitor vital physiological signals, human behaviors, and the environment, and advanced algorithms that detect anomalies from the acquired sensor data. More recently, advancements in artificial intelligence (AI) and sensing technology have empowered more efficient extraction and delivery of valuable healthcare information to patients and providers.

To explore the latest developments and challenges in sensor-based information extraction for disease diagnosis and health monitoring, this Special Issue compiles nine original research articles and two reviews providing new insights into the development of novel sensors and associated data processing algorithms.

One group of articles focuses on innovative sensors/systems for obtaining specific signals for healthcare applications. For example, Glowinski et al. [1] designed a system for the acquisition and processing of bioelectric signals from electromyographic sensors to operate a brushless DC motor (BLDC). Their findings demonstrated that the BLDC motor’s rotation speed and direction could be modulated based on the EMG signals from the biceps brachii and triceps muscle groups. This research concluded that the developed system was scalable and allowed users to adjust signal levels. The developed technology showed promise for use in areas such as rehabilitation, exoskeletons, and prostheses. Another study by Metshein et al. [2] reported a novel method to evaluate the penetration of chemically and biologically active compounds from curative mud through human skin using electrical bioimpedance (EBI) analysis. A specialized gold-plated electrode was designed for this purpose. The research applied mud packs and tap-water compresses to the forearms of volunteers and compared the results with measurements from dry skin. Initial findings from their pilot study involving ten volunteers indicated that EBI was a promising tool for monitoring skin electrical properties.

Another group of articles is related to the processing algorithms of signals or images acquired by wearable sensors or other sensing modalities. Jambhale et al. [3] investigated several significant biomarkers collected by a chest-worn device, called RespiBAN Professional, for stress detection in substance use disorder (SUD). The effective biomarkers to detect stress were determined to include those based on electrodermal activity, body temperature, and body accelerometry. This research also differentiated between mental and physical stress, and evaluated the effects of meditation, aiming to apply these findings to SUD treatment in the future. The goal of this work was to develop an integrated portable system capable of detecting and managing SUD using wearable biosensors. Felix et al. [4] introduced an algorithm designed for wearable electrocardiographic devices to accurately detect heartbeat intervals, with robustness against noise and featuring low computational complexity. The proposed algorithm fitted a least-squares parabola to the ECG signal, adaptively shaping it to detect the peaks of the R-wave. Evaluation on the QT and BIH-MIT databases showed the algorithm’s substantial advantages over classical methods and competitiveness with state-of-the-art approaches.

Besides wearable sensors, Mazurek et al. [5] explored the effectiveness of impulse-radar and depth sensors for monitoring elderly individuals in healthcare settings. Feedforward and recurrent neural networks were used to integrate the sensor data, and the performance was evaluated against a reference method. Both neural networks were trained on synthetic data and their accuracy was compared in terms of field-acquired parameters, such as the number of turns made while walking, distance traveled, and average walking speed. The results indicated that the nonlinear autoregressive network with exogenous inputs outperformed the other method. As a new, non-invasive ultrasound imaging technique, shear wave elastography (SWE) allowed for the assessment of muscle’s mechanical properties. Palac et al. [6] investigated the intra-rater reliability of SWE for measuring respiratory muscles during tidal breathing in adolescent athletes. The findings showed that the diaphragm measurements were more reliable than those from the intercostal muscles regardless of the respiratory phases and probe positions. Excellent reliability was found for the diaphragm shear modulus at the peak of tidal expiration in the transverse probe position. This study suggested that the technique was potentially useful for muscle examination in athletes. 

Public datasets were also analyzed to extract embedded information and used to evaluate and/or compare algorithms. Shi et al. [7] designed a novel transformer-based network architecture, called TCU-Net, for retinal vessel segmentation in optical coherence tomography angiography (OCTA) images. It addressed the limitations of traditional convolutional networks by introducing an efficient cross-fusion transformer module and a channel-wise cross-attention module. TCU-Net was tested on the Retinal OCTA Segmentation (ROSE) dataset, showing high accuracy and AUC values on different subsets (ROSE-1 and ROSE-2), indicating its superior performance and robustness in vessel segmentation compared to existing state-of-the-art methods. Mou et al. [8] developed a computational framework to link the morphological features of cell nuclei with RNA expressions using microscopic histology images and RNA-sequencing data of 456 breast tissue samples from the Genotype-Tissue Expression Project. Their categorization of samples based on nucleus morphology and biological pathway analysis revealed that variations in RNA expression were associated with differences in breast tissue morphology. This approach provided a detailed view of RNA expression related to specific imaging features in healthy breast tissue and suggested potential applications for understanding RNA morphology connections in other tissues and organs. Khodadadi et al. [9] presented Patient Forest, a novel end-to-end approach for learning data representations from electronic health records (EHRs), with the aim of predicting the risk of readmission and mortality. Experiments conducted on MIMIC-III and eICU datasets demonstrated that Patient Forest is an accurate and reliable classifier. This research showed that Patient Forest outperformed existing machine learning models, particularly when training data were limited. 

The third group of papers reviews the application of machine learning techniques for disease monitoring and management. An et al. [10] conducted a study exploring the utilization of machine learning techniques in the healthcare domain. They highlighted the potential of these techniques in enhancing diagnostic and treatment capabilities for medical professionals. This paper offered a comprehensive overview of both supervised and unsupervised machine learning algorithms and the evaluation of their accuracy. Another review by Palavicini et al. [11] focused on how artificial intelligence can be utilized to identify health issues, enhance overall health, and prevent severe patient damage. Their research aimed to assess the progress of medicine with intelligent sensor-based devices, identify commonly used devices in medical practices, determine the most benefited population, and explore the current applications of AI technology by physicians. 

In summary, this Special Issue of *Sensors* entitled “Sensing-Based Biomedical Communication and Intelligent Identification for Healthcare” will provide the readers with a rich view of the latest prospects of sensors for healthcare applications.
